# Penicillin Acylase-Catalyzed Effective and Stereoselective Acylation of 1-phenylethylamine in Aqueous Medium using Non-Activated Acyl Donor

**Published:** 2010-04

**Authors:** D.T. Guranda, G.A. Ushakov, V.K. Švedas

**Affiliations:** Belozersky Institute of Physicochemical Biology, Faculty of Bioengineering and Bioinformatics, Lomonosov Moscow State University, Russia

**Keywords:** stereoselective enzymatic acylation in aqueous medium, direct condensation, enantiomerically pure compounds, penicillin acylase

## Abstract

Until recently, the biocatalytic preparation of enantiomerically pure amines was based on stereoselective acyl transfer in an organic medium using activated acyl donors. The possibility of performing an effective and enantioselective enzymatic acylation of amines in an aqueous medium without using activated acyl donors was demonstrated for the first time as the example of direct condensation of phenylacetic acid and racemic 1–phenylethylamine. Direct condensation of the acid and the amine took place at mild reaction conditions with a high initial rate (3.3 µmole/(l·h)), degree of conversion (80% acylation of active amine enantiomer), and enantioselectivity (enantiomeric excess of the product was more than 95%). The suggested approach has remarkable advantages compared to enzymatic reactions in organic media and is of practical value for the biocatalytic preparation of enantiomerically pure compounds at mild conditions using readily available reagents.

## INTRODUCTION

Since enzymes demonstrate such unique characteristics as chemo–, regio– and stereospecificity, biocatalytic processes have significant advantages as compared to traditional organic synthesis. The advantages of enzymatic technologies are especially noticeable during the synthesis of multifunctional or enantiomerically pure compounds. Experts relate further possibilities of growth in the enantiomerically pure compound market (which has shown an annual growth rate of more than 13% in the last decade [2]) mainly to the introduction of biocatalytic technologies [1]. The synthesis of enantiomerically pure amines is of especial interest, since these compounds are important chiral synthones in the pharmaceutical and agrochemical industries [3].

Currently, most of the industrial processes involving biocatalysis are based on the hydrolysis of amides and esters, although synthetic reactions are often of more practical use [4]. This is based on the fact that in the most favorable aqueous medium for biocatalytic processes the equilibrium of the reaction is shifted towards hydrolysis. In order to shift the equilibrium towards the formation of amide and ester bonds, most sources in the literature suggest to perform the reactions in various organic solvents [5]. For a long time, enzymatic reactions in organic media have been considered to be the most promising approach for these synthetic reactions [6–9]. Therefore, researchers focused their attention on the use of enzymes that were stable in organic solvents. Until recently, the possibility of enzymatic acylation of amines had been demonstrated only in non–aqueous water–free organic media with the use of lipases as catalysts of the acyl transfer reaction from the activated acyl donors to the amines [10–12]. Even though the use of lipases has been fairly successful, biocatalysis in non–aqueous media has a number of complications, the most obvious being low enzymatic activity [13], as well as the necessity to control the use of ecologically toxic organic solvents, which constitute the bulk of pharmaceutical industrial waste [14].

A critical analysis of studies in the field of stereoselective biocatalytic transformations has indicated that at sufficient optimization the enzymatic synthesis in aqueous media could be much more effective than was previously thought. Among other examples, this was demonstrated by the highly effective and stereoselective acylation of amines due to penicillin acylase–catalyzed acyl transfer in 100% aqueous solution [15]. This demonstration was made possible thanks to the unique catalytic traits of the penicillin acylase from * Alcaligenes faecalis * [16]; namely, its high catalytic activity and stability under alkaline conditions (pH ~ 10), which were optimal for the acylation of basic amines.

This report presents the first results of an approach for the effective and stereoselective enzymatic acylation of amines in an aqueous medium by direct condensation of an amine and a carboxylic acid, which is used as an acyl donor. An effective approach for the isolation of α –phenylethylamine enantiomers is of practical importance, since they are used as resolving agents and chiral auxiliaries during the production of enantiomers of various classes of chemical compounds [17, 18].

## MATERIALS AND METHODS

**Reagents**. This study used phenylacetyl chloride (Sigma, USA); phenylacetic acid (Aldrich Chemie, Germany); (*R*)– and (*S*)– α –phenylethylamine (Fluka, Switzerland); phenylmethylsulphonylfluoride, sodium dodecyl sulphate (Merck, Germany); acetonitrile («CryoChrome», Russia); * N* –phenylacetyl derivatives of α –phenylethylamine were synthesized according to [15]; the penicillin acylase from * Alcaligenes faecalis * was supplied by the LLC Innovations and Higher Technologies of MSU (Russia). The concentration of penicillin acylase active sites was determined according to [16].

**HPLC analysis**. Concentrations of the reaction mixture components and the enantiomeric purity of the synthetic reaction product ( * N* –phenylacetyl–(*R*)– α –phenylethylamine) were measured by HPLC using a Perkin Elmer Series 200 (Perkin Elmer, USA) equipment as described earlier [15].

**The direct enzymatic condensation of phenylacetic acid and (±)–** α **–phenylethylamine** was performed with equimolar amounts of reagents (0.2 М ), which were constantly mixed in a thermostated cell of a pH–stat (Titrino 718, Metrohm, Switzerland) at pH 7.5 and 15^о^ С ; the penicillin acylase concentration was 12 µM. The reaction product, *N* –phenylacetyl–(*R*)–phenyletylamine, precipitated in the course of enzymatic reaction. Aliquots (50 μl) of the heterogeneous reaction mixture were added to 1.95 ml of the mobile phase in order to fully solubilize the reaction components and to stop the reaction. These samples were then diluted with eluent and analyzed using standard and chiral HPLC. The enzymatic reaction continued until it reached an equilibrium state; that is until the concentrations of the reaction components reached fairly constant values. The reaction product was then filtered onto a glass filter, washed with water, recrystallized from aqueous ethanol, and then dried in a desiccator over a layer of calcium chloride. Yield 0.145 g, (38%); e.e. 95%; mp 117–118°C; 1H NMR (250 MHz, CDCl3): 1.29 (d, 3H, CH3), 3.47 (s, 2H, CH2), 5.01 (m, 1H, CH), 5.49 (d, 1H, NH), 7.04–7.29 (m, 10H, Ph). MS m/z: 239 (62, M), 120 (49, PhCH2CH(NH)CH3), 105 (100, PhCCH3), 91 (75, PhCH2), 77 (68, Ph), 65.

## RESULTS AND DISCUSSION

Stereoselective acylation is the key step in the resolution of racemic amines, and the use of enzymes as catalysts of this process seems to be a very promising approach. However, it is worth noting that primary amines are strongly basic compounds, and that they can be effectively acylated only in alkaline aqueous solutions (pH approximately 10) or a water–free organic medium, where the majority of enzymes exhibit diminished catalytic activity and low stability. Also, conducting enzymatic reactions in organic solvents requires the use of activated acyl donors, which can spontaneously acylate reactive amino groups in a non–stereoselective manner, thus lowering the enantiomeric purity of the reaction product [3, 9]. We suppose that these drawbacks can be circumvented by conducting the enzymatic acylation of amines in an aqueous medium using the direct condensation of the carboxylic acid and the amine. In this case, thermodynamically favorable conditions for the condensation reaction are achieved in a practically neutral medium (pH approximately 6–8), where most enzymes are highly active and stable. Both reacting substrates are highly soluble and stable at these conditions, which allow the use of highly concentrated solutions of the reagents and creates favorable thermodynamics for enzymatic amine acylation. The driving force of the reaction, which makes the whole process so efficient, is the shift of equilibrium towards synthesis, caused by the precipitation of the poorly soluble reaction product. Notably, direct condensation does not require activation of the acyl donor, which simplifies the enzymatic amine acylation process and reduces costs. The wide substrate specificity and stereoselectivity of penicillin acylases towards N–acylated derivatives of amino compounds [16, 19, 20] provides reason to hope that enzymatic amine acylation via direct condensation and using this family of enzymes will be effective.

**Fig. 1:**
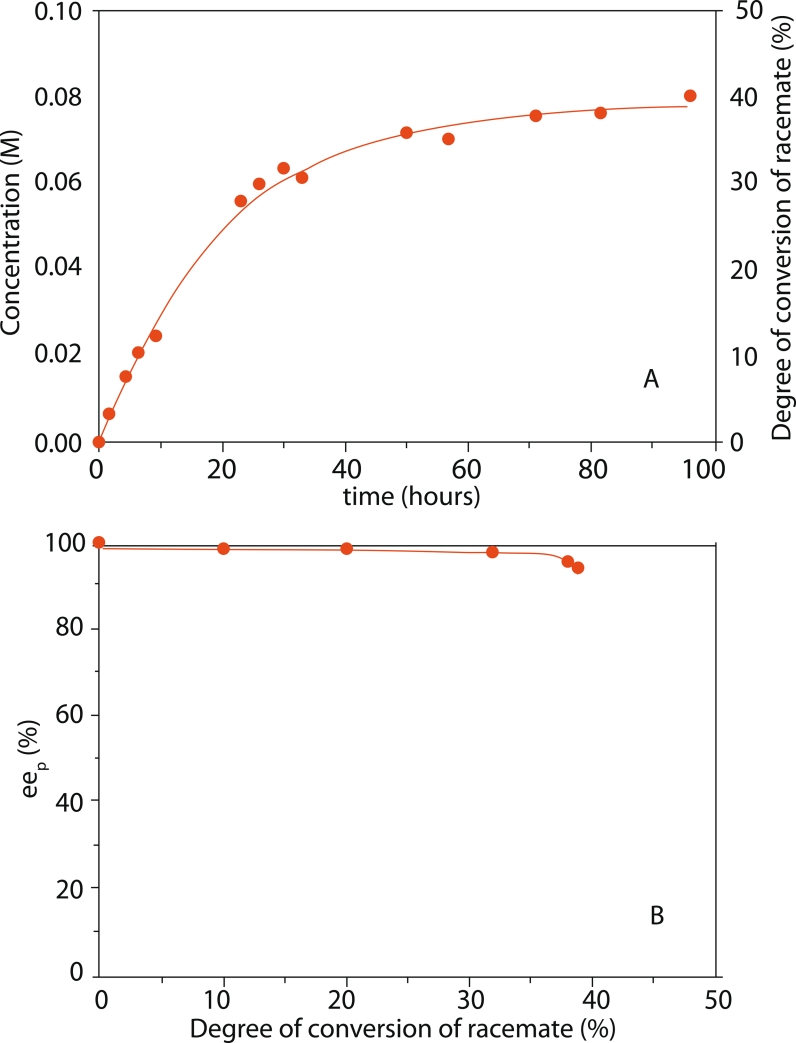
Synthesis of enantiomerically pure *N*-phenylacetyl-(*R*)-phenylethylamine using direct stereoselective condensation of phenylacetic acid and a (±)-α-phenylethylamine racemic mixture in an aqueous medium catalyzed by penicillin acylase obtained from Alcaligenes faecalis: (A) – The integral kinetics of product formation, (B) – Enantiomeric excess of the target product at several degrees of conversion. The conditions are described in the Methods section

Indeed, the first experiments show that the condensation of phenylacetic acid with a racemic α –phenylethylamine catalyzed by penicillin acylase in an aqueous medium is highly effective, with an initial rate of 3.3 mmol /(l·h). Only a few minutes after the beginning of the reaction, the target product (*N* –phenylacetyl–(*R*)– phenylethylamine ) starts precipitating. The synthesis progresses quite rapidly until it reaches a 30–35 % degree of conversion of the initial reagents’ concentrations (Fig.1). The condensation reaction then slows down, which is probably caused by the thermodynamic equilibrium being achieved. This is confirmed by the control experiment, which shows that the enzyme is not inactivated at this point, and that the penicillin acylase retains virtually all of its initial catalytic activity.

The analysis of the enantiomeric purity of the target product at different stages of conversion validates (Fig.1, B) a high stereoselectivity of enzymatic acylation. The enantiomeric excess of the target product is 98% and 96% at conversion degrees of 30% and 40%, respectively. After the reaction stops, the precipitated *N* –phenylacetyl–(*R*)–phenylethylamine can be easily isolated from the reaction mixture. The yield of active enantiomer of *N* –phenylacetyl–(*R*)–phenylethylamine is 80 %, and the enantiomeric excess is 95 %.

The amine acylation method described here has none of the drawbacks of enzymatic reactions conducted in organic solvents, and it is of practical use for the biocatalytic production of enantiomerically pure compounds under mild conditions out of readily available reagents. In order to assess the perspectives of this method, it is necessary to compare it with enzymatic acylation of amines in an aqueous medium using activated acyl donors, as proposed previously [15]. Both approaches have their advantages and drawbacks. The use of activated acyl donors provides a high reaction rate and depth of acylation, as well as a high enantiomeric purity of the target product. However, it involves the use of more expensive acyl donors and takes place in an alkaline medium, which makes the enzyme less stable. The most important advantage of the direct condensation method is the possibility of using inexpensive acyl donors and milder reaction conditions, in which most enzymes, including the whole penicillin acylase family, are more active and stable. These advantages may prove decisive for the use of direct condensation during preparative resolution of a wide range of racemic amines.

The results presented in this report are just the first observations, and the process of enzymatic amine acylation by direct condensation should be further studied and optimized. It is likely that stereoselective enzymatic acylation of amino compounds by direct condensation will become an important integral part of the general biocatalytic method for preparation of enantiomerically pure amino compounds [21]. In further research, we plan a detailed study of the various factors affecting the equilibrium of the reaction and a careful analysis of the kinetics of penicillin acylase–catalyzed stereoselective acylation reactions by direct condensation in an aqueous medium.

## CONCLUSIONS

This is the first report on the possibility of effective and enantioselective enzymatic acylation of primary amines in an aqueous medium without the use of activated acyl donors. Direct condensation catalyzed by penicillin acylase is highly efficient, and the target product of high enantiomeric purity can be easily isolated from the reaction mixture with a high yield. This method can be used for preparative biocatalytic synthesis of enantiomerically pure amines under mild reaction conditions using readily available substrates and a biocatalyst.
